# InterPro in 2019: improving coverage, classification and access to protein sequence annotations

**DOI:** 10.1093/nar/gky1100

**Published:** 2018-11-06

**Authors:** Alex L Mitchell, Teresa K Attwood, Patricia C Babbitt, Matthias Blum, Peer Bork, Alan Bridge, Shoshana D Brown, Hsin-Yu Chang, Sara El-Gebali, Matthew I Fraser, Julian Gough, David R Haft, Hongzhan Huang, Ivica Letunic, Rodrigo Lopez, Aurélien Luciani, Fabio Madeira, Aron Marchler-Bauer, Huaiyu Mi, Darren A Natale, Marco Necci, Gift Nuka, Christine Orengo, Arun P Pandurangan, Typhaine Paysan-Lafosse, Sebastien Pesseat, Simon C Potter, Matloob A Qureshi, Neil D Rawlings, Nicole Redaschi, Lorna J Richardson, Catherine Rivoire, Gustavo A Salazar, Amaia Sangrador-Vegas, Christian J A Sigrist, Ian Sillitoe, Granger G Sutton, Narmada Thanki, Paul D Thomas, Silvio C E Tosatto, Siew-Yit Yong, Robert D Finn

**Affiliations:** 1 European Molecular Biology Laboratory, European Bioinformatics Institute (EMBL-EBI), Wellcome Trust Genome Campus, Hinxton, Cambridge CB10 1SD, UK; 2 School of Computer Science, The University of Manchester, Manchester M13 9PL, UK; 3 Department of Bioengineering & Therapeutic Sciences, University of California, San Francisco, CA 94158, USA; 4 European Molecular Biology Laboratory, Structural and Computational Biology Unit, Meyerhofstr.1, 69117 Heidelberg, Germany; 5 Swiss-Prot Group, SIB Swiss Institute of Bioinformatics, CMU, 1 rue Michel-Servet, CH-1211 Geneva 4, Switzerland; 6 Medical Research Council Laboratory of Molecular Biology, Francis Crick Avenue, Cambridge Biomedical Campus, Cambridge CB2 0QH, UK; 7 J. Craig Venter Institute (JCVI), 9605 Medical Center Drive, Suite 150, Rockville, MD 20850, USA; 8 Center for Bioinformatics and Computational Biology, University of Delaware, Newark, DE, USA; 9 Biobyte Solutions GmbH, Bothestr 142, 69126 Heidelberg, Germany; 10 National Center for Biotechnology Information, National Library of Medicine, NIH Bldg, 38A, 8600 Rockville Pike, Bethesda, MD 20894, USA; 11 Division of Bioinformatics, Department of Preventive Medicine, University of Southern California, Los Angeles, CA 90033, USA; 12 Protein Information Resource, Georgetown University Medical Center, Washington, DC, USA; 13 Department of Biomedical Sciences, University of Padua, via U. Bassi 58/b, 35131 Padua, Italy; 14 Department of Agricultural Sciences, University of Udine, via Palladio 8, 33100 Udine, Italy; 15 Fondazione Edmund Mach, Via E. Mach 1, 38010 S. Michele all’Adige, Italy; 16 Structural and Molecular Biology, University College London, Darwin Building, London WC1E 6BT, UK

## Abstract

The InterPro database (http://www.ebi.ac.uk/interpro/) classifies protein sequences into families and predicts the presence of functionally important domains and sites. Here, we report recent developments with InterPro (version 70.0) and its associated software, including an 18% growth in the size of the database in terms on new InterPro entries, updates to content, the inclusion of an additional entry type, refined modelling of discontinuous domains, and the development of a new programmatic interface and website. These developments extend and enrich the information provided by InterPro, and provide greater flexibility in terms of data access. We also show that InterPro's sequence coverage has kept pace with the growth of UniProtKB, and discuss how our evaluation of residue coverage may help guide future curation activities.

## INTRODUCTION

Technological advances, coupled with dramatic reductions in sequencing costs in recent years, have enabled a revolution in nucleic acid sequencing. Researchers are now able to sequence entire genomes or determine millions of environmentally derived sequences over the course of a single experiment. Such accomplishments, previously prohibitive in terms of cost and achievable only in collaboration with large sequencing centres, are now relatively routine. As a result, the scientific community is dealing with an enormous and expanding deluge of sequence data encoding millions of proteins that have not yet been experimentally characterized, nor probably ever will be.

To address this situation, functional annotation of the vast majority of protein sequences relies on the automatic transfer of information from a few experimentally characterized sequences onto a set of homologues. By far the largest source of automatic annotation of sequences in the UniProt Knowledgebase (UniProtKB) (1) (the central hub of protein sequences) is InterPro. Launched in 1999, InterPro is derived from 14 different specialist member databases: CATH-Gene3D (2), the Conserved Domains Database (CDD) (3), HAMAP (4), PANTHER (5), Pfam (6), PIRSF (7), PRINTS (8), ProDom (9), PROSITE Patterns (10), PROSITE Profiles (10), SMART (11), the Structure–Function Linkage Database (SFLD) (12), SUPERFAMILY (13) and TIGRFAMs (14). These databases use diagnostic models (profile hidden Markov models (HMMs), other forms of profiles, position-specific scoring matrices, and regular expressions, collectively known as ‘signatures’), against which protein sequences can be searched to assign potential functions.

Each InterPro member database has a different area of expertise, and collectively they largely offer complementary levels of protein classification, ranging from broad-level (e.g. classifying protein domains into superfamilies) to comparatively granular assignments (a protein is a member of a specific family, or possesses a particular type of domain or site). In addition, a subset of the InterPro member databases are potentially able to assign amino acid residue-level annotation, including key catalytic residues and those that are involved in ligand binding: these are CATH-Gene3D, HAMAP, Pfam, PIRSF, PROSITE, CDD and SFLD, although only the latter two currently have this facility enabled in InterPro.

InterPro also provides additional information about sequence features, such as consensus annotation of long-range intrinsic disorder (provided by MobiDB-lite, a derivative of the MobiDB database (15)) and prediction of signal peptides, transmembrane regions and coiled-coils, via the SignalP, Phobius, TMHMM and Coils software packages (16–19). Integrating all of these data together, InterPro offers highly comprehensive and in-depth functional annotation of protein sequences.

InterPro and its associated software are widely disseminated and utilised by the scientific community, and the database is recognised as a key community data resource (20) (https://www.elixir-europe.org/platforms/data/core-data-resources). New InterPro releases are made available for public download every two months. InterPro data are also used by a variety of other annotation pipelines, including Ensembl (21), Ensembl Genomes (22), PDBe (23), BLAST2GO (24), Genome Properties (https://www.ebi.ac.uk/interpro/genomeproperties/), PhytoPath (25), MEGAN (26) and MGnify (previously known as EBI Metagenomics) (27). The InterProScan web services, meanwhile, provide analysis of user-submitted sequences, processing in excess of 40 million sequence searches per month.

The largest application of InterPro data is their import into UniProtKB, where InterPro annotations provide the foundation for automatic annotation of proteins. To enable UniProtKB to perform this annotation task, InterPro matches are calculated on a monthly basis via the InterProScan software package (28), ensuring new sequences are annotated.

## RESULTS

### Updates to InterPro content

Member database signatures are not added into InterPro automatically, but undergo a manual inspection and integration process. Matches between the signatures and the latest version of UniProtKB are evaluated to ensure no known false positives are present. Signatures that represent the same biological entry are integrated together into individual InterPro entries, reducing redundancy (e.g. the CDD, PROSITE Profile, Pfam and SMART signatures representing the CUB domain (cd00041, PS01180, PF00431, SM00042, respectively) are integrated into a single InterPro entry (IPR000859)). New InterPro entries are manually annotated with a name, a descriptive abstract and Gene Ontology (GO) terms (29) that can be consistently assigned to all proteins matched by that entry. Hierarchical relationships are identified between evolutionarily related InterPro entries, tracing those that represent smaller, functionally specific subfamilies of larger families, or subclasses of broader classes of domain. The annotation and sequence match information is reviewed monthly, following the UniProtKB match calculation update, and InterPro entry annotation is updated based on any revised sequence information or biological knowledge (e.g. if a previously uncharacterised protein has been ascribed a particular function). This requires substantial curation effort, but is nevertheless vital in order to maintain annotation accuracy given the evolving nature of the underlying data (30).

InterPro regularly incorporates member database updates and new signatures. Details of InterPro releases and the member database updates integrated into the resource since our last report (31) are given in Table 1. Of particular note is InterPro release 61.0, which included an update to PANTHER 11.0. This had numerous changes compared to PANTHER version 10.0, including a switch to using HMMER3 as the underlying sequence analysis algorithm. While this update transformed the speed at which PANTHER matches could be calculated in InterPro, it however carried the risk of potentially losing ∼3000 existing InterPro entries, as their underlying signatures had been substantially modified as part of the database rebuild between PANTHER 10.0 and 11.0. Therefore, as part of InterPro release 61.0, an extensive curation effort was dedicated to locating the most appropriate signatures that could replace the modified PANTHER signatures (either in PANTHER 11.0 or from other member databases), ultimately resulting in the loss of only 190 at-risk InterPro entries.

**Table 1.** Member database versions integrated into InterPro since release 61.0

InterPro release	Member database update
61.0	SFLD (2), PANTHER (11.1)
62.0	CATH-Gene3D (4.1), HAMAP (201701.18), PROSITE patterns (20.132), PROSITE profiles (20.132)
63.0	Pfam (31.0)
64.0	CDD (3.16)
65.0	SFLD (3), PANTHER (12.0)
66.0	HAMAP (2017_10), PROSITE patterns (2017_09), PROSITE profiles (2017_09)
67.0	CATH-Gene3D (4.2)
68.0	HAMAP (2018_03), PROSITE patterns (2018_02) and PROSITE profiles (2018_02)
69.0	(MobiDB-lite update)
70.0	SFLD (4)

InterPro has added 5320 net new entries in the last two years, representing an overall increase of 18%. These new entries were based on 7013 new member database signatures that have been integrated into the resource, with 1693 signatures added to existing entries. In total, InterPro now comprises 35 020 entries based on 48 938 signatures. This has expanded InterPro's coverage of UniProtKB sequences from 79.8% (InterPro release 60.0) to 80.9% (InterPro release 70.0) (see Table 2). Whilst this may seem a small increase in percentage terms, the improvement in coverage should be evaluated in context with the substantial growth of the underlying sequence database; UniProtKB concurrently increased from ∼71 million sequences to ∼125 million. Thus, InterPro's relatively small coverage increase represents significant progress.

**Table 2.** Coverage of UniProtKB by InterPro signatures

Sequence database	Number of proteins in database	Number of proteins with one or more matches to InterPro
UniProtKB/reviewed	558 125	539 742 (96.7%)
UniProtKB/unreviewed	124 797 108	100 920 355 (80.9%)
UniProtKB (total)	125 355 233	101 460 097 (80.9%)

**Figure 1.**
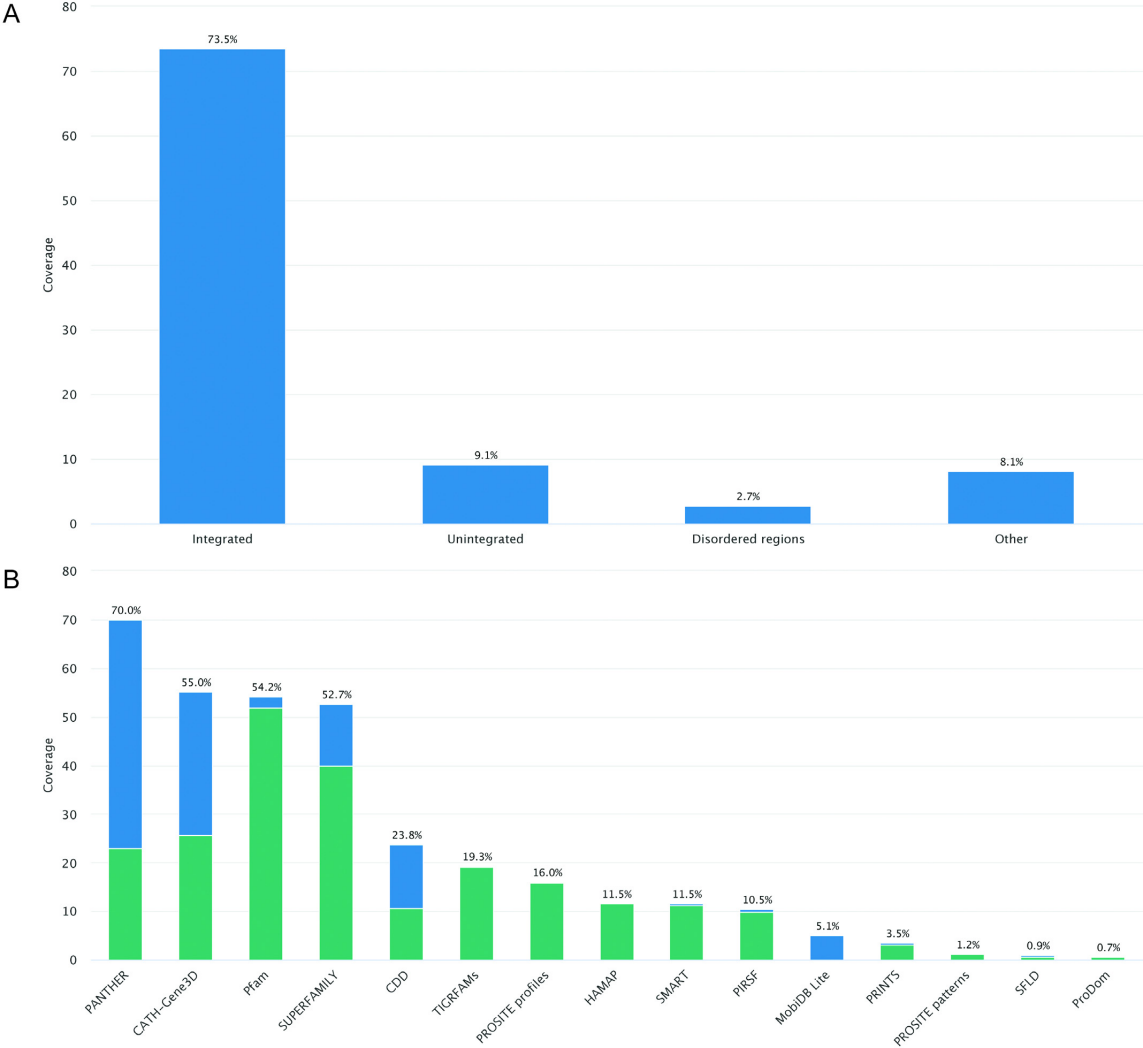
InterPro coverage of amino acid residues in UniProtKB. (**A**) Unique residue coverage of UniProtKB by signatures integrated into InterPro, member database signatures awaiting integration, intrinsically disordered regions, and regions predicted to be signal peptides, transmembrane domains or coiled-coils. (**B**) Residue coverage of InterPro's contributing member databases. Residues matched by signatures integrated into InterPro are shown in green, and residues found only in signatures not yet integrated are shown in blue.

In addition to sequence coverage, we have also assessed the amino acid residue coverage of InterPro and its member databases. In Figure 1A, we show the cumulative unique residue coverage of: (i) InterPro entries (27.7 out of 37.7 billion residues, i.e. 73.5%); (ii) signatures provided by the member databases awaiting integration into InterPro (9.1%); (iii) residues that are found in intrinsically disordered regions (2.7%) and (iv) residues that are found in other sequence features annotated by InterPro, such as coiled-coil, transmembrane regions and signal peptides (8.1%). This means that 93.4% of UniProtKB residues receive some level of annotation and leaves a total 6.6% (or 2.5 billion residues) that are yet to be annotated by InterPro and/or its associated member databases.

The value added by aggregating the member database is shown by comparing the above numbers to the contributions made by each member database (Figure 1B). PANTHER provides the greatest residue coverage, as may be expected given the size of the database (in terms of profile HMMs) and its focus on representation of full-length protein families. Databases such as Pfam, SUPERFAMILY and CATH-Gene3D offer similar levels of residue coverage to each other, but as expected, this is lower than PANTHER, as they focus on discrete domains. Meanwhile, smaller databases, including those focusing on active or binding sites, or short motifs that confer functional specificity, provide detailed functional annotation but inevitably offer the least overall residue coverage.

### A new InterPro programmatic interface and associated website

InterPro release 70.0 was accompanied by an entirely new website (https://www.ebi.ac.uk/interpro/beta/) aimed at providing greater flexibility in querying, presenting and retrieving data. One of the primary drivers behind the architectural design of the website was also the provision of an Application Programming Interface (API) that would be utilized by both the website (client) and users directly accessing the data.

### The API

The API is designed around a Representational State Transfer (REST) framework, with requests structured as URLs and responses in JSON (JavaScript Object Notation) format. The general structure of an API URL query is to combine attributes in order to define both the main data type returned by the API and any filters to be applied to the dataset.

There are currently six main API endpoints, each corresponding to a key data type in InterPro: Entries, Proteins, Structures, Sets, Proteomes and Taxonomies. The Entries endpoint provides access to data pertaining to InterPro and member database entries. Protein data are imported from UniProtKB. Structures are imported from the SIFTS (32) mapping provided by PDBe. The Sets endpoint provides access to groupings of Entries. This latter feature is new to InterPro and enables the representation of concepts such as Pfam clans and CDD superfamilies/collections. Data for Proteomes and Taxonomies are imported from UniProtKB, and linked to Entries through their matched Proteins. The Taxonomies endpoint allows users to list all InterPro entries or member database signatures that have matches to particular taxonomic lineages, or to create subsets of data based on this information (for example, retrieving only the mammalian sequences from all proteins that InterPro classifies as members of a particular family). Finally, the Proteomes endpoint is also a new addition to InterPro, and indicates whether a protein is a member of the UniProtKB Proteomes collection (i.e. derived from an isolate organism whose genome has been completely sequenced).

**Figure 2.**
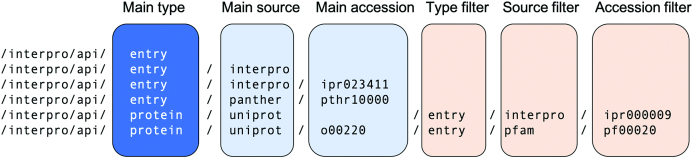
Example API queries. From top to bottom, the first example returns a count of the total number of entries in InterPro and its member databases. The second retrieves information on all InterPro entries. The third and fourth examples return information specific to InterPro entry IPR023411 and PANTHER entry PTHR10000, respectively. The fifth returns InterPro information for all UniProtKB sequences matching InterPro entry IPR00009. The final request returns details of the match between Pfam entry PF00020 and UniProkKB sequence O00220. Further details about the structure of the API URLs are given in (Supplementary Data Table S1).

The general principle for structuring a URL query is to specify the main data type (endpoint) to be returned, followed by one or more filters and endpoints. The secondary filters/endpoints act to either limit the data to particular source databases and/or accessions, or to define extra information from the other endpoints to be combined with the dataset (see Figure 2 for examples). Online documentation for the API is available at https://www.ebi.ac.uk/interpro/beta/help/documentation/.

### The website

The new website is implemented as a user-interface to the API, allowing querying and filtering of data through a feature-rich set of web components developed with the open source React/Redux framework. A number of new features have been added, most notably the Browse page. Here, users can explore, search and filter the Entries, Proteins, Structures, Taxonomies, Proteomes and Sets data types. A key aim of the new design is to promote the InterPro member databases. As such, the browse view shows a list of InterPro and member databases on the left and a set of data type-specific filters along the top. These filters change depending on the data type and member database selections. For example, the ‘Integrated Database’ filter is only shown when viewing Entries and only if InterPro is selected from the member database selection list. By default, results in the browse page are presented in tabular form, but users have the option of browsing summary information of most data types as a grid, or, in the case of Taxonomy, as a navigable tree.

**Figure 3.**
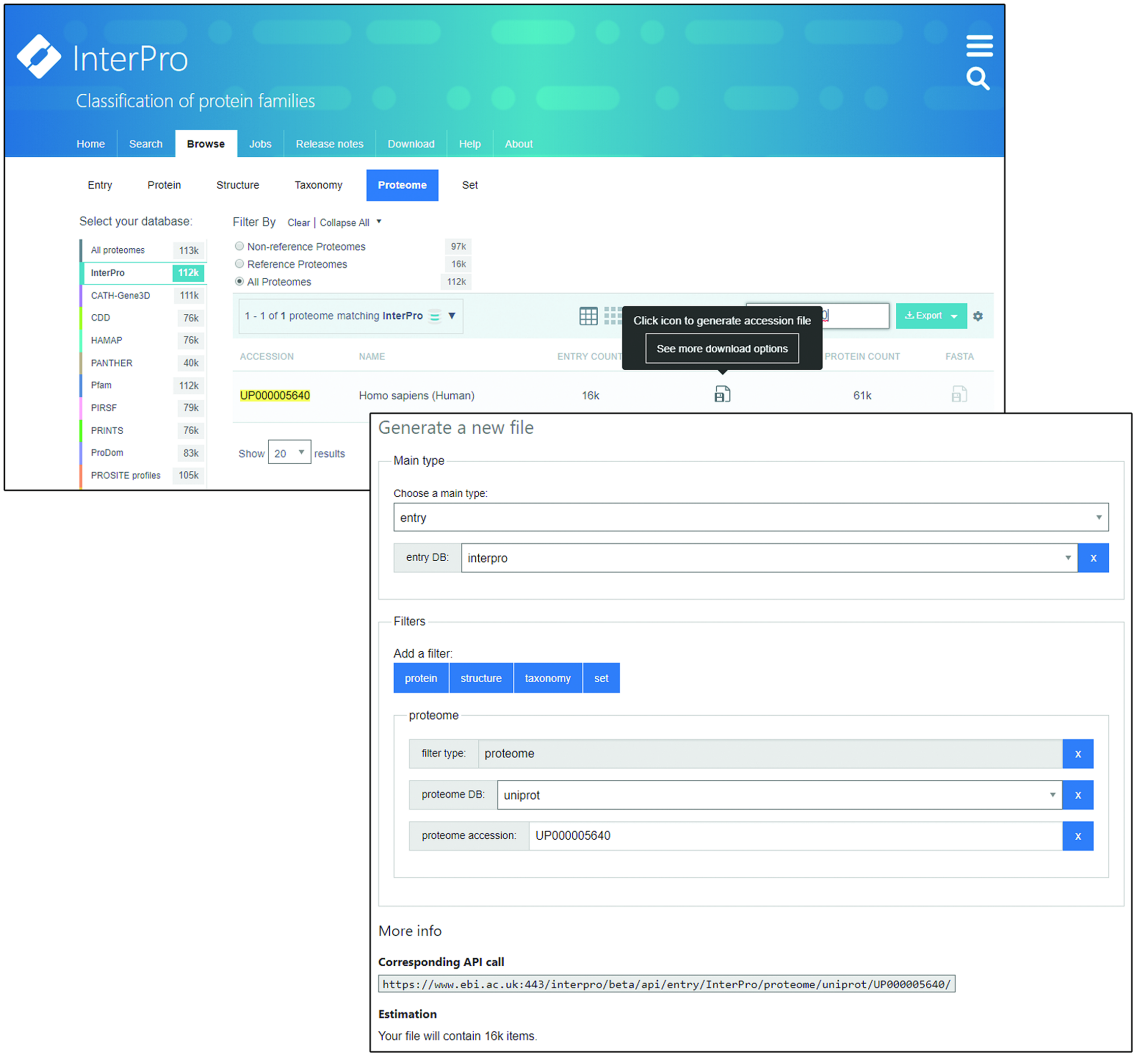
Selecting data to download from the Browse page creates a link to an appropriately pre-filled form and API request on the Download page.

While increasing the search capability, we have also implemented new options for downloading data and for running InterProScan. The Download page allows users to select data types, apply filters and select their required download format. In addition to the Download page, rows in the tabular view component for taxonomy data contain links allowing download of entry accessions or protein sequences in FASTA format. These links also show a pop-up link directing users to the new Download page, pre-filling the download form according to the data required (Figure 3). Owing to the potential size of some downloads, the number of sequences that can be downloaded into a single file has been restricted. For large files, users are directed to the Download page for that file, which dynamically generates code snippets in JavaScript, Python and Perl to enable the user to download the selected data programmatically via the API.

As mentioned above, the new website utilizes a series of web components to display different data. For example, representations of protein sequences in the Protein pages, Structure pages and in the domain architectures section of the Entry pages use an extended version of ProtVista (33) to display sequence match positions. The ProtVista component supports dynamic scaling, from viewing the whole sequence down to the level of residues. Similarly, we have embedded and adapted the LiteMol viewer (34) to enable 3-dimensional (3D) visualization of entries and structures. The LiteMol and ProtVista components have been linked on the Structure pages to enable users to highlight regions on 3D representations of protein structures corresponding to the ProtVista linear representation of families and domains.

We have also re-used the taxonomy browser component originally developed for the HMMER website (35). This component allows users to browse through a taxonomy tree, whereby information is dynamically loaded based on the part of the tree being visualized. As parts of the tree can be very information dense, the library implements a fish-eye view to aid readability. As the taxonomic information is typically combined with other data - for example, viewing the organisms belonging to the taxonomic phylum Chordata that have matches to the SH2 domain (IPR000980) - the tree is reduced to only those branches containing information matching the combined query; branches that lack matches are not shown.

Finally, the submission of protein sequences and viewing the InterProScan results have been integrated more tightly into the new website. The status of searches can be viewed and managed in the Jobs page, and results can be viewed using the same dynamic tools available in the Protein pages of the website.

### A new InterPro entry type: homologous superfamily

As part of the integration process, InterPro curators classify entries into types (families, domains, repeats or sites) depending on the biological entity they represent. Family and domain entries are placed into distinct, non-overlapping hierarchies, with domain entries able to occur in the same hierarchy as other domains, but not within the same hierarchy as family entries, and vice versa. Overall, this system works well, as the majority of member databases use single signatures to represent families or domains that are relatively stable over time such that their sequence matches do not usually change significantly.

However, the CATH-Gene3D and SUPERFAMILY databases adopt a different approach, using collections of underlying HMMs per entry, which is necessary to encapsulate diverse structural families. Furthermore, as new related but diverse structures are incorporated, additional HMMs may be added to the same entry. As a result, when either of these databases is updated, there can be considerable flux in the sequence matches for a given entry. Furthermore, as both CATH-Gene3D and SUPERFAMILY update asynchronously (and are thus updated asynchronously in InterPro), the relationships of some entries to each other and to other entries in InterPro can be difficult to maintain. Consequently, following an update to CATH-Gene3D or SUPERFAMILY, there is often a period of de-integration in InterPro, where entries from these databases are removed before being re-annotated and re-inserted into the hierarchy, based on the new sets of sequences they match.

To help resolve this situation, we created a new entry type, ‘*homologous superfamily*’, in InterPro 65.0, representing signatures that match proteins sharing a common evolutionary origin, as indicated by their structural similarities. *Homologous superfamily* type entries have a relaxed threshold for integration, in that they are not manually curated into hierarchies. Instead, their relationships to other InterPro entries are calculated entirely automatically, based on the intersection of their matched sequence sets, without additional biological contextual data that would usually be considered by a curator.

**Figure 4.**
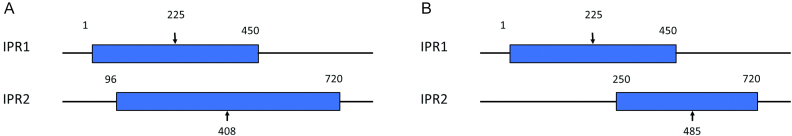
Intersecting (A) and non-intersecting (B) InterPro matches for the purpose of calculating homologous superfamily relationships.

**Figure 5.**
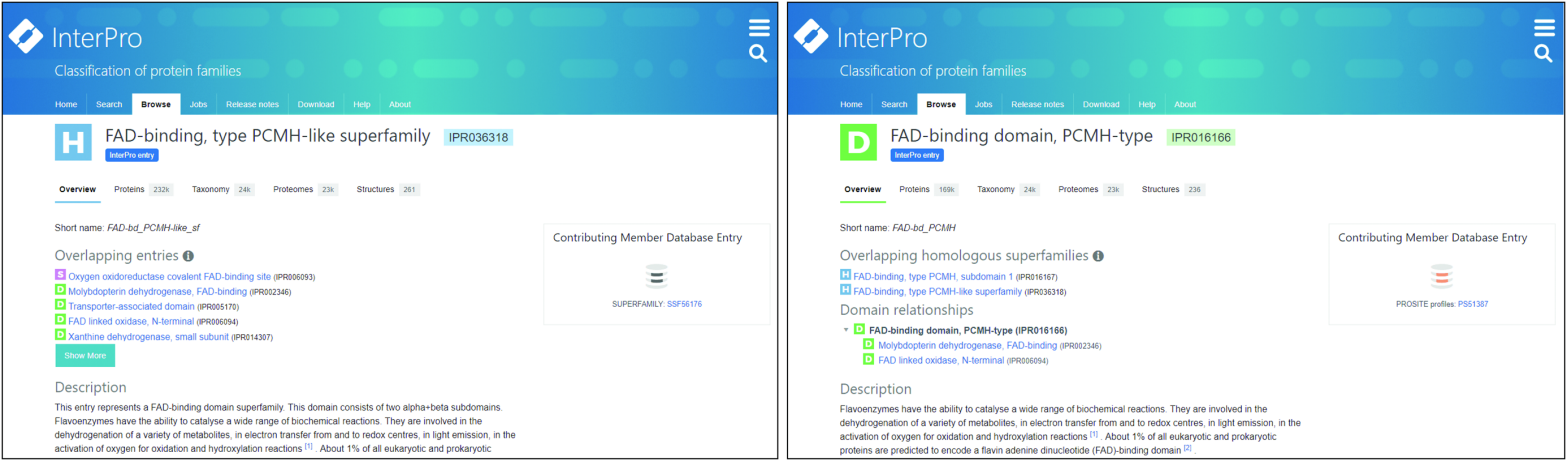
Reciprocal ‘overlapping homologous superfamilies’ and ‘overlapping entries’ links on the *homologous superfamily* entry (left) and other InterPro entry (right) pages which display the relationships between these entry types.

Under this system, *homologous superfamilies* and InterPro entries are examined to see if their sequence matches intersect (defined as where the midpoint of the match of one entry to a sequence lies between the match boundaries of the other entry (see Figure 4)). Each pair of *homologous superfamily* and InterPro entry found to overlap by this criteria are then evaluated for relatedness. The Jaccard index and Jaccard containment index (36) for the pair are determined. If either of these indices is >0.75 (a threshold chosen because it gives a relatively robust approximation of manually curated relationships), it is assumed that the *homologous superfamily* and the InterPro entry are related to each other. These relationships are calculated at every InterPro release, and unlike the curated parent-child relationships that are generated for other entries, these relationships are listed on the ‘overlapping entries’ and ‘overlapping homologous superfamilies’ sections on the *homologous superfamily* entry or other InterPro entry pages, respectively (see Figure 5).

**Figure 6.**
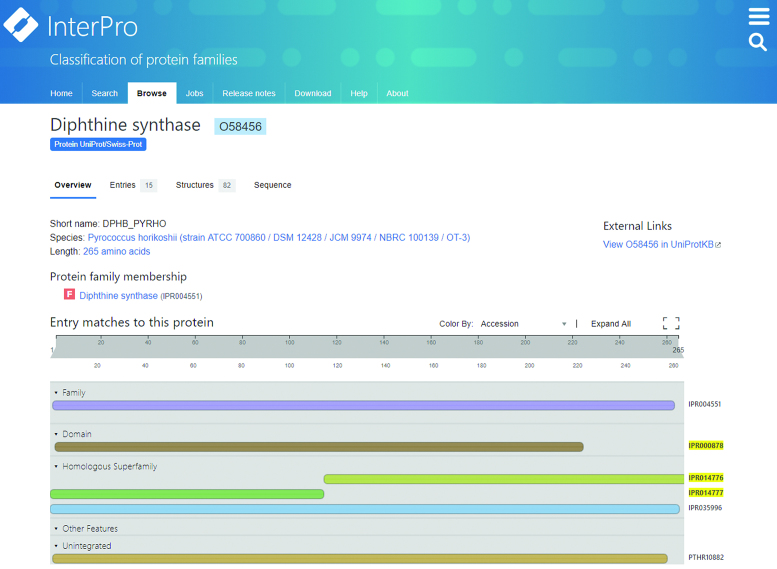
The *homologous superfamilies* annotation track on the ProtVista view on the proteins page allows structural information to be placed in context with other annotations.

*Homologous superfamilies* are assigned their own annotation track on the InterPro protein overview page. This allows users to place the structural components of proteins in context with other sequence features, such as functional domains or active sites. The automatic relationship calculation, meanwhile, provides an added advantage, in that it makes *homologous superfamilies* easier to place in context with other InterPro data. An example to illustrate this point is the tetrapyrrole methylase domain, which comprises two subdomains. One subdomain is composed of a three-layer(αβα) sandwich, the second subdomain is composed of a two-layer sandwich. Pfam provides a signature (PF00590, integrated into InterPro entry IPR000878) that spans the whole domain (i.e. the two sub-domains), while CATH-Gene3D provides entries that identify the two structurally distinct subdomains (CATH-Gene3D entries G3DSA:3.40.1010.10 and G3DSA:3.30.950.10, integrated into IPR014777 and IPR014776, respectively) (see Figure 6). Under InterPro's previous entry type and annotation rules, there was no mechanism to formally link the domain and subdomain entries to each other, as they could not be integrated into the same InterPro entry, nor were they considered to have a parent-child relationship in the InterPro hierarchy due to differences in length. However, under the new *homologous superfamily* entry type, this relationship is captured automatically.

The addition of the *homologous superfamily* entry type and automated reconstruction of their relationships to other InterPro entries have enabled greater flexibility and efficiency in terms of integrations into InterPro. This has allowed the integration of more CATH-Gene3D and SUPERFAMILY entries into the resource than ever before (3748 collectively, compared to 3137 in release 60.0). As a result, links between the relatively few known structures and many more protein sequences are now more extensive and evident within the resource.

### Discontinuous domains

The Pfam, CATH-Gene3D and SUPERFAMILY databases all provide information about discontinuous domains, where a domain may comprise two or more segments that are separate from each other along the linear sequence, but form a single globular domain in 3D space. This type of information, derived from structural and/or bounded domain data, was not previously modeled in InterPro, resulting in a discrepancy in matches (where InterPro counted each segment as a separate match, compared to a single match in the member database) or where the disrupted domain could mask another domain nested between the two discontinuous segments.

**Figure 7.**
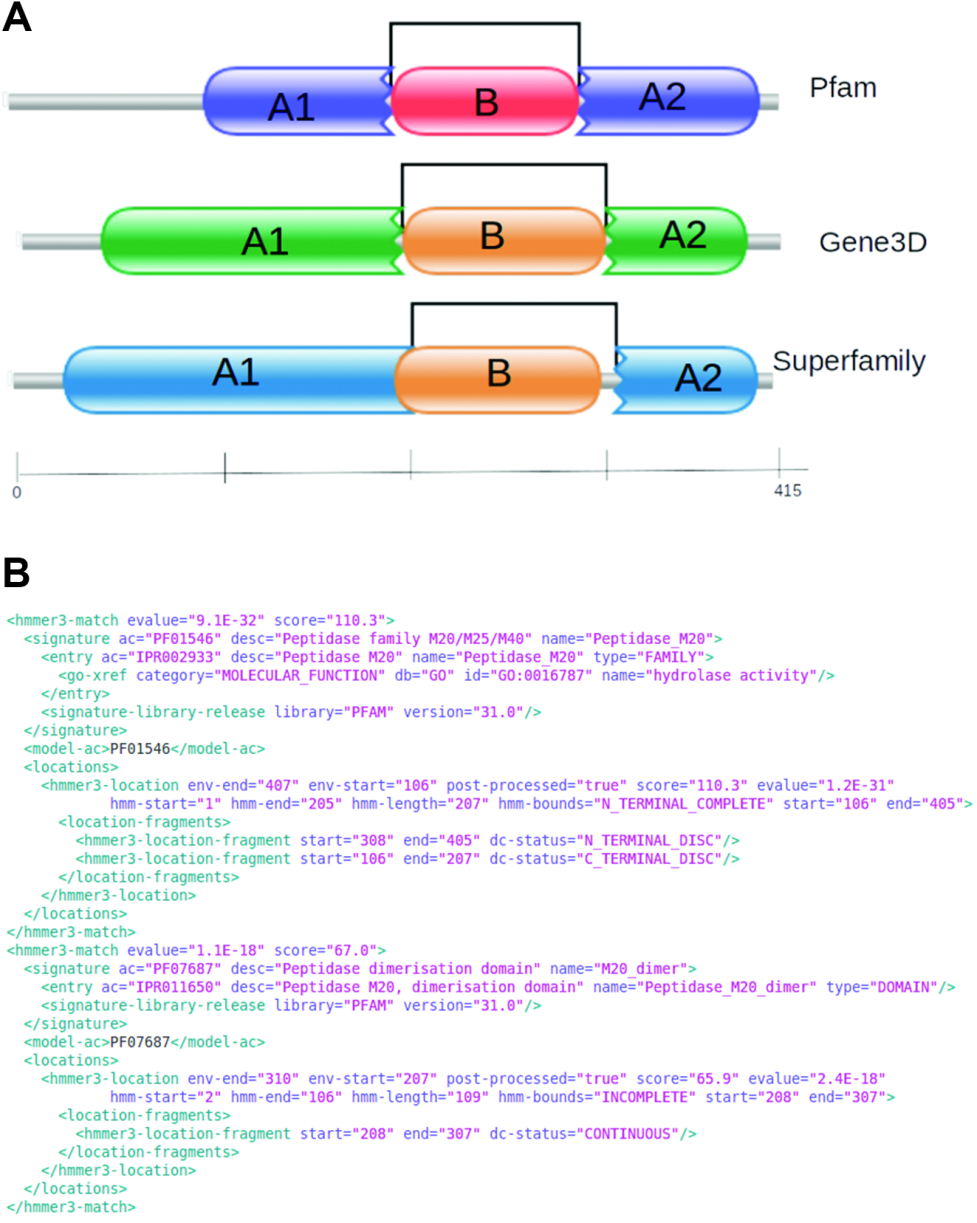
(**A**) Pfam, CATH-Gene3D and SUPERFAMILY domain matches for UniProtKB sequence A0A0Q0BJI4. The segments A1 and A2 form a discontinuous domain and segment B is an independent nested domain. (**B**) Example InterProScan XML output for the Pfam matches shown in (A).

Representation of discontinuous domains was added to InterPro in release 70.0, and there are currently 2635 discontinuous domains signatures, providing matches to ∼17 million UiProtKB proteins. To enable this representation, the InterProScan post-processing algorithms were updated to analyse annotation of fragmentary matches, along with bounding information showing whether a match was discontinuous in the N- or C-terminal direction, or in both directions. This information can now be found in the InterProScan output, and is also displayed graphically on the InterPro protein overview page (see Figure 7).

### Extended Intrinsic disorder annotations

MobiDB-lite was incorporated into InterPro release 60.0, providing the ability to annotate long-range disordered regions using the resource. As part of InterPro release 70.0, we have updated to MobiDB-lite version 1.5 (15), which adds classification of sub-regions of the overall disordered region according to the sub-region properties: positive polyelectrolytes, negative polyelectrolytes, polyampholytes, polar, cysteine-rich and proline-rich (15). This level of annotation is important, because different conformational ensembles have been associated with different types of disorder. For example, it has been shown that strong polyampholytes have a preference for random coil or more compact conformations, depending on charge segregation (37). Weak polyampholites are found in more compact conformations (e.g. the first 100 residues on the protein α-synuclein (38)), while negative and positive polyelectrolytes both tend to be found in random coil ensembles (37). However, both tendencies seem to be only valid for regions longer than 30 residues and with a relatively low proline content (39). Furthermore, some of these classes have been found to specialize in different functions in the cell (40). Proteins containing strong polyelectrolytes play different structural roles depending on their net charge. For example, positive polyelectrolyte regions are preferentially found in ribosomal proteins, while negative polyelectrolyte regions are used by eukaryotes in the cytoskeleton (40). Polyampholytes seem to be used by bacteria, archaea and eukaryotes in the biosynthesis of cellular assets, such as macromolecular complexes (40). Thus, these additional annotations provided within InterPro enable an even deeper understanding of the potential roles of the intrinsic disorder predictions.

## DISCUSSION

Maintenance of annotation coverage and accuracy are key challenges in light of burgeoning volumes of sequence data. Poised to enter its 20th year, InterPro continues to meet these challenges through the combined hard work of its member databases and its own substantial curation and production efforts. Consequently, the resource has not only kept pace with the growth of UniProtKB, but has increased its coverage over the last two years, despite an expansion in the number of underlying protein sequences by over 75%.

Analysis of InterPro's coverage of UniProtKB amino acid residues shows that, considering all types of InterPro-derived information (signature matches, disordered regions and predicted sequence features), <7% of residues currently lack any form of annotation. Within that 7% of unannotated residues, there remains the challenge to the InterPro member databases to determine those that represent novel protein families or domains, those that are outliers of existing families and those that may be protein sequence mis-predictions.

Although large numbers of CATH-Gene3D, CDD and SUPERFAMILY signatures are yet to be integrated, it is worth noting that these will not significantly change InterPro's residue coverage, as many of the residues annotated by these resources are already represented by other databases. Nevertheless, these remain a priority for integration, as they provide either functionally specific annotation or a mechanism to link large numbers of sequences with no known structure to the few solved structures. We expect that the new *homologous superfamily* type will significantly accelerate this process for CATH-Gene3D and SUPERFAMILY.

The PANTHER database currently provides the greatest unique coverage of residues not yet represented in InterPro (6.4%). Incorporating as many PANTHER entries that provide additional residue coverage will ensure that we maximize the contribution of this database. To achieve this, we will continue to streamline curation efficiency (for example, exploring the use of Jaccard index-based systems to guide curation of InterPro entry types other than *homologous superfamilies*).

The range of annotations in InterPro is also expanding through new developments, such as the *homologous superfamily* entry type, discontinuous domain annotations and expanded intrinsic disorder predictions. To better serve this extended and growing data, we have made a number of improvements to the resource. These include development of our new API and website, which strive to make a richer set of InterPro data readily accessible in a flexible manner. Such developments will help us better serve our user community, both now and in the coming years.

## Supplementary Material

Supplementary Data
