# Supplementary material for: InterPro in 2019: improving coverage, classification and access to protein sequence annotations
Source: Nucleic Acids Res. 2018 Nov 6;47(Database issue):D351–60. doi: 10.1093/nar/gky1100 (PMC6323941; doi:10.1093/nar/gky1100)
Supplement: Supplementary Data [file gky1100_supplemental_files.pdf]

SUPPLEMENTARY DATA

API URL Structure

Endpoint blocks are the most important aspect of a query to the InterPro API. An endpoint block can be composed of up to 3 parts: an endpoint type (e.g., entry), a source database (e.g., InterPro) and a unique identifier (e.g., IPR00009). The endpoint type is mandatory, while the other two parts are optional.

The presence of the different parts of the main block define the filter and determine the type of response returned by the API. The following rules determine the response type:

- A query that only contains the endpoint type will return a list of aggregated values giving the total counts of all the unique entities, grouped by each data source of that type. For example, a query '/protein' will return a JSON object with the number of proteins in UniProtKB, including the reviewed and unreviewed sections.
- When the query includes a data source, the response is a paginated list of the entities that belong to that source, including some basic information. For example, the entities returned by the query '/structure/pdb' include the name, accession and experiment type for each PDB structure.
- If an accession block is part of the request, the response contains more detailed information about the requested entity. For example, the API response to the query 'protein/UniProt/P50876' will be a JSON object containing all the available information for that protein that has been imported into InterPro, including its sequence, description, *etc*.

Requests can contain more than one endpoint block, so each of the results of the main block can be filtered by any other endpoint. In this way, a query such '/entry/protein/reviewed' defines the main endpoint as entry. As it doesn't include a data source or an accession, the response will return a count of how many entries exist per source database (InterPro, CATH-Gene3D, CDD, and so on). Since we have a second endpoint block in the URL ("reviewed"), the query will be restricted (in this case to just the reviewed proteins).

Additional functionality can be achieved by using the type-specific attributes outlined in Table S1. For example, the list of InterPro entries that represent domains can be obtained using the query '/entry/InterPro?type=domain'. This mechanism allows extension of the API, while conserving the general structure of the RESTful URL.

**Endpoint**	**Source database(s)**	**Type-specific attributes**	**Notes**
Entry	InterPro, CATH-Gene3D, CDD, HAMAP, PANTHER, Pfam, PIRSF, PRINTS, ProDom, PROSITE profiles, PROSITE patterns, SFLD, SMART, SUPERFAMILY, TIGRFAMs	annotation	allows association of information, such as alignments
		go_term	filter based on GO terms
		go_category	filter based on GO category
		integrated	filter based on integration status
		type	filter by entry type (family, domain, *etc*)
Protein	UniProtKB	ida	group by InterPro Domain Architecture
		residues	show per-residue annotation
		tax_id	filter by taxonomy
		protein_evidence	filter by protein evidence
		go_term	filter by GO term
		match_presence	filter by database hit
Structure	PDB	resolution	filter by resolution
		experiment_type	filter by experiment type
Set	Pfam, CDD	-	-
Proteome	UniProtKB	is_reference	filter by reference proteomes
Taxonomy	UniProtKB	-	-

Table S1. An overview of the endpoints in the InterPro API showing the databases and type-specific attributes that can be incorporated into REST requests.
